# Effects of Tai Chi and Qigong intervention on anxiety and stress in diabetic and hypertensive Brazilian patients: a randomized controlled trial

**DOI:** 10.31744/einstein_journal/2025AO1076

**Published:** 2025-03-17

**Authors:** Laís Renata Almeida Cezário Santos, Anderson Taíra, Rosana de Fátima Possobon, Marcelo de Castro Meneghim, Chien-Lin Su, Paola Lavin, Soham Rej, Gláucia Maria Bovi Ambrosano, Karine Laura Cortellazzi

**Affiliations:** 1 Universidade Estadual de Campinas Faculdade de Odontologia de Piracicaba Department of Health Sciences and Children's Dentistry Piracicaba SR Brazil Department of Health Sciences and Children's Dentistry, Faculdade de Odontologia de Piracicaba, Universidade Estadual de Campinas, Piracicaba, SR Brazil; 2 Sociedade Brasileira de Tai Chi Chuan e Cultura Oriental Piracicaba SR Brazil Sociedade Brasileira de Tai Chi Chuan e Cultura Oriental, Piracicaba, SR Brazil; 3 McGill University Department of Epidemiology, Biostatistics and Occupational Health Montreal Quebec Canada Department of Epidemiology, Biostatistics and Occupational Health, McGill University, Montreal, Quebec, Canada; 4 McGill University Jewish General Hospital The Lady Davis Institute Montreal Quebec Canada Department of Psychiatry, The Lady Davis Institute, Jewish General Hospital, McGill University, Montreal, Quebec, Canada

**Keywords:** Tai ji, Qigong, Perceived stress scale, Anxiety, Hypertension, Diabetes mellitus, Primary health care, Outcome assessment, health care, Stress, psychological, Middle-aged, Aged, Complementary therapeutic methods

## Abstract

**Objective:**

This study investigated the effects of Tai Chi/Qigong practice over 13 weeks on anxiety and perceived stress levels in middle-aged or older Brazilian hypertensive and diabetic individuals assisted in a primary healthcare setting.

**Methods:**

Ninety-one patients with *diabetes mellitus* and/or hypertension diagnosed and followed up at a primary care center were randomized to the Tai Chi/Qigong intervention (n = 45) or waitlist (n = 46) group for 13 weeks. Outcome measures were collected at baseline and at 6 and 13 weeks for anxiety (State-Trait Anxiety Inventory [STAI]) and stress (Perceived Stress Scale - PSS14). The primary endpoint of the study was 13 weeks. We used a linear mixed-effects model to analyze the primary and secondary outcomes, considering the treatment group and time as covariates and treating the subject as a random effect.

**Results:**

Of the included 91 participants, 53 completed the 6-week assessment, and 42 completed the 13-week assessment. After 13 weeks, the Tai Chi/Qigong Group had lower scores for anxiety (STAI Estimate = -6.421; SD = 2.679; 95% confidence interval (95%CI) = [-11.615,-1.224]; p = 0.018) and stress (PSS14 Estimate = -9.290; SD= 2.262; 95%CI= [-13.678,-4.906]; p<0.001).

**Conclusion:**

A 13-week Tai Chi/Qigong intervention was efficacious in lowering anxiety and perceived stress scores in middle-aged and older patients with *diabetes mellitus* and hypertension and can potentially be used in primary care centers as an adjunct therapy.

**Trial registration:** Brazilian Registry of Clinical Trials (ReBEC), Identifier: U1111-1276-1787.

## In Brief

A 13-week Tai Chi/Qigong intervention reduced anxiety and stress in middle-aged and older Brazilian patients with diabetes and hypertension. With significant improvements in the State-Trait Anxiety Inventory and PSS14 scores, this practice shows potential as an effective adjunctive therapy in primary care.

## Highlights

A 13-week Tai Chi/Qigong program significantly reduced anxiety and stress in Brazilian patients with diabetes and hypertension.

Tai Chi/Qigong offers an effective complementary therapy to improve health outcomes in primary care settings.

This study highlights the potential of Tai Chi/Qigong for enhancing mental and physical well-being in chronic disease management.

## Graphical abstract

**Figure:**
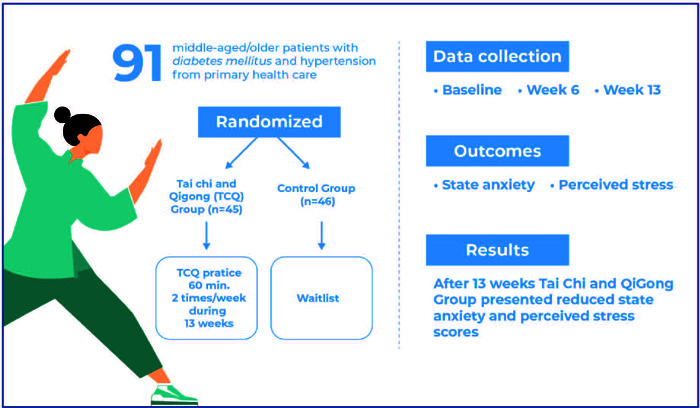

## INTRODUCTION

*Diabetes mellitus* (DM) and hypertension (HTN) are the most common noncommunicable diseases (NCDs), primarily affecting middle-aged and older adults in low- and middle-income countries.^(1,2,3)^ Their widespread occurrence is a global concern, with approximately 422 million^(2)^ and 1.28 billion^(3)^ individuals affected by DM and HTN, respectively. Together, these conditions account for 74% of all global deaths, in addition to disability and decreased quality of life.^(1,4)^

People with chronic conditions such as DM and HTN encounter challenges that impact both their physical and emotional well-being, increasing the likelihood of experiencing psychological symptoms and mental health issues, such as anxiety and stress.^(5)^ Approximately 68.7% of individuals with chronic diseases exhibited symptoms of stress, whereas 51.1% experienced anxiety.^(5)^ Anxiety and stress are associated with long-term glycémie and blood pressure variability,^(6,7,8)^ treatment non-adherence, low self-care, increased risks of cardiovascular disease, disability, stroke, mortality, social difficulties, elevated use of health services, and financial burden.^(6,7,8,9,10,11,12)^ Conventional treatment for anxiety and stress (pharmacotherapy, psychotherapy, or both)^(13,14)^ can be difficult to access because of diverse barriers, such as cost, stigma, logistics, tolerability, and the high risk of adverse effects.^(14,15,16)^ Hence, there is a need for effective alternative interventions to improve the mental health outcomes and well-being of individuals with chronic diseases.^(17)^

Tai chi (TC) and Qigong (QG) are the most common meditative practices based on traditional Chinese medicine principles that combine physical and mental training through meditation, self-healing, and self-cultivation exercises.^(18,19)^ Both TC and QG are considered safe, meditative-based mind-body practices that combine low and coordinated body posture and movements with deep rhythmic breathing, meditation, and mental focus.^(19,20,21,22,23)^ They are used to achieve body strength (essential for self-defense and fighting), promote physical and mental well-being, and prevent and treat illnesses.^(19)^ These two practices are often combined and known as Tai Chi/Qigong (TCQ) or “Tai Chi Easy” in practices and studies involving older and/or inactive adults due to their gentler movements that are easy to perform and repeated for easy learning.^(19,24,25)^

Evidence suggests that TC and QG, independently or in combination (TCQ), may improve the clinical outcomes of diabetes and hypertension by controlling blood pressure^(22,26,27,28,29,30)^ and glycemic levels.^(31,32,33,34)^ Some studies have investigated the effect of TC and QG practiced independently on the mental health of patients with DM and HTN;^(35,36,37)^ however, the authors did not conduct these studies in the primary healthcare context and community setting. In addition, only one pilot study^(38)^ investigated the effect of TCQ on the mental health of middle-aged and older adults (women); however, not all participants presented with DM and/or HTN.

In Brazil, a middle-income country, the prevalence of individuals diagnosed with DM and HTN who experience stress and anxiety remains high.^(4,39)^ Hence, there is an urgent need for efficacious, accessible, and non-pharmacological interventions to address psychological comorbidities and aid in preventing the risks of disability, mortality, and reduced quality of life.^(4,39)^ Furthermore, primary healthcare is the main gateway to the Brazilian Unified Health System for people diagnosed with HTN and DM, where they receive interventions to promote health and prevent and treat diseases in their community.^(40)^ In recent years, the Brazilian Ministry of Health has given importance to implementing primary healthcare complementary and integrative medicine practices that can be adjunct therapies to conventional treatment and improve the physical and mental well-being of patients.^(41)^ However, more studies are needed to prove the effects of complementary practices implemented in the community,^(41)^ such as TCQ, in this population.

Specifically, we hypothesized that after 13 weeks of TCQ practice, middle-aged and older individuals with DM and hypertension would show improved anxiety (STAI) and perceived stress (PSS14) scores.

## OBJECTIVE

The effects of Tai Chi/Qigong sessions over 13 weeks can effectively manage anxiety and stress in middle-aged or older Brazilian patients with *diabetes mellitus* and hypertension, followed by primary healthcare.

## METHODS

### Study design

We conducted a randomized, two-arm, waitlist-controlled, parallel-group trial. The study protocol was approved by the Research Ethics Committee of *Faculdade de Odontologia de Piracicaba, Universidade Estadual de Campinas* (FOP-UNICAMP) (CAAE: 33386620.3.0000.5418; # 5.486.918). All the participants provided written informed consent. Consort guidelines were followed in this study.

### Participants

The participants were recruited from a primary healthcare center in Brazil and screened in person. Eligible participants signed a written informed consent form prior to the intervention. Recruitment took place from 06/01/2022 to 07/30/2022, and follow-up took place from 08/01/2022 to 10/29/2022.

### Inclusion criteria

We included participants ≥50 years old, of both sexes, diagnosed with DM (fasting blood glucose ≥200)^(42)^ and/or HTN (systolic blood pressure ≥140 mmHg and diastolic blood pressure ≥90mmHg)^(43)^ by a clinician and in treatment for at least one year at the primary care center, and provided informed consent.

### Exclusion criteria

Individuals with medical conditions that could limit their capacity to consent or participate in the study (such as severe hearing impairment, recent surgery, vision impairment, or neurological impairment) were excluded. In addition, participants with serious musculoskeletal diseases, limb amputation, severe heart, respiratory, renal, or liver disease, and cancer were excluded. Additionally, those currently involved in any mind-body exercise modality (*e.g.,* TCQ, yoga, or pilates) were excluded.

### Randomization and blinding

A total of 102 candidates were screened and 91 individuals with DM and/or HTN were assigned to either an intervention Group (TCQ training) or a Control Group (waitlist) in a 1:1 ratio at a Brazilian primary healthcare center. An independent researcher used a computer-based randomizer to perform random allocation. Participants and assessors were not blinded to the allocation.

### Interventions

#### TCQ Group

The TCQ Group (n=45) participated for 13 weeks, with one hour of TCQ classes twice weekly (120 min/week) delivered by a certified TCQ instructor. The classes were delivered to a community of participants next to the primary healthcare centers where they received follow-up. During the classes, the participants learned the principles and concepts related to TCQ and performed warm-ups (10 min), Qigong (30 min), Tai Chi Yang-style (10 min), and relaxation (5 min) movements. The Qigong movements performed were the Wu Chi pose, Post pose, crane exercise, tree hugging, goose flight, swimming in the air, caressing clouds, side stretching, and gentle Buddha's hands. The TC movements performed were as follows: preparatory form, initial form, stroking of the bird's tail, simple whipping, raising hands, taking a step, white heron extending its wings, defending the knee to the left, and pushing. At the end of the program, participants learned eight TC movements chosen from a long series of Yangstyle Tai Chi. Participants received videos (through WhatsApp) of the TCQ movements to practice at home between sessions. During the intervention period, participants continued their routine habitual care for HTN or DM control (diet, medication, medical, and other professional consultations).

### Control Group

The Control Group (n=46) continued with their habitual routine (diet, physical exercise, medication, blood glucose, and blood pressure monitoring) and usual care (including clinical consultations and health promotion actions provided for multidisciplinary teams in primary health care centers to control and maintenance of health) for DM and HTN control. These participants were placed on the waitlist and only received TCQ classes after the end of the trial.

### Data collection procedures

Data were collected in a primary healthcare center in the participants' community at different times: at baseline (TO), after 6 weeks (T1), and after 13 weeks (T2). Data were collected through interviews (with illiterate participants) and self-reported questionnaires (with literate participants).

### Outcome measures

#### Primary outcomes

To evaluate anxiety (State Component of the State-Trait Anxiety Inventory [STAI] scores), the participants answered the STAI scale, a 20-item self-report questionnaire that measures anxiety levels that is well-validated for the Brazilian population.^(44,45)^ The 4-point Likert scale can be used to examine an individual's reaction to stressful situations and emotions at a given time.^(44,45)^ The total STAI scores range from 20-30 (mild anxiety), 31-49 (moderate anxiety), and 50-80 (severe anxiety).^(44,45)^ The primary endpoint was the score obtained at 13 weeks.

#### Secondary outcomes

To evaluate perceived stress (Perceived Stress Scale-PSS14 scores), the participants completed the PSS-14 to assess their perceived stress level.^(46,47)^ This 14-question instrument, validated for the Brazilian population, uses a 5-point Likert scale to measure how individuals perceive their life events as stressful.^(47)^ This scale predicts health-related outcomes from one to two months prior.^(46,47)^ The PSS14 scores range from 0-14 (no stress), 15-28 (mild stress), 29-42 (moderate stress), and 43-56 (severe stress).^(46,47)^ The secondary endpoint was the score obtained at 13 weeks.

### Sociodemographic data, habits, and medical history

#### Demographics and medical history

Through an interviewer-administered questionnaire, we collected information regarding sociodemographic data (age, sex, income, ethnicity, occupation, education, type of residence, marital status, religion), health status, and habits (physical activity habit, physical activity frequency, and physical activity duration in the past months), feelings of anxiety and/or depression, type of chronic disease (DM, HTN, or both), medication intake (anxiolytic/anti-depressive, anti-diabetic, antihypertensive drugs), smoking habits, alcohol drinking behavior, whether they were following a diet for DM/HTN control, and perceived health.

### Clinical information

Anthropometric parameters (height and weight), arterial blood pressure (systolic and diastolic), and fasting blood glucose (FBG) levels were measured at baseline, at 6 weeks (T1), and at 13 weeks (T2). All measurements were taken in succession, in a quiet location, by the same nurse, with calibrated equipment prior to data collection, and during the same time of the day.

### Anthropometric data

Participants wearing light clothing and without shoes were weighed on a digital scale (measured to the nearest 0.1kg), and their height was measured using a stadiometer. Their body mass index (BMI) was calculated using the formula BMI = kg/m^2^ (weight in kilograms divided by [height in meters]^2^) using Microsoft Excel software.

### Fasting blood glucose

The participants were instructed to fast for 8 h before blood glucose collection. Fasting blood glucose levels were measured using a finger stick to collect blood samples and a blood glucose analyzer (On Call® Plus glucometer, ACON Biotech, China).

### Arterial blood pressure (systolic and diastolic)

The blood pressure of the participants was measured after 15 min of rest, with the participant seated, by the auscultatory method of the left brachial artery, using a stethoscope (Premium Stethoscope, Ningbo, China) and a sphygmomanometer (Premium Aneroid Sphygmomanometer, Wenzhou, China).

### Sample size calculation

The sample size (n=91) was determined using G*Power software.^(48)^ The target effect size was set to 0.25, considering the limited number of previous studies. A total required sample size of 91 was calculated by applying F-tests based on an effect size = 0.25 (average effect), as reported previously,^(49)^ a significance level = 0.05, power (1-*β*) = 0.80, number of groups = 2, and number of repeated measurements = 3.

### Statistical analysis

Baseline sociodemographic and clinical outcomes were summarized using counts and percentages for categorical variables and means and standard deviations for continuous variables. Baseline information was compared between the TCQ and Control Groups using the χ^2^ and *t*-student t-tests to assess the balance of baseline covariates between the treatment groups. Based on the strategy of the intent-to-treat analysis, linear mixed-effects models were considered for the primary and secondary outcomes (measured at T0, T1, and T2). The treatment groups (TCQ and Control), time (T0, T1, and T2), and interaction terms (treatment groups by time) were considered as covariates, treating the subject as a random effect. Moreover, owing to the high dropout rate, a per-protocol analysis approach was considered based on the same linear mixed-effects model. Statistical analyses were performed using R (Version 3.5, R Foundation for Statistical Computing, Vienna, Austria).^(50)^

## RESULTS

### Study enrolment, intervention adherence, and participant characteristics

**Figure 1:**
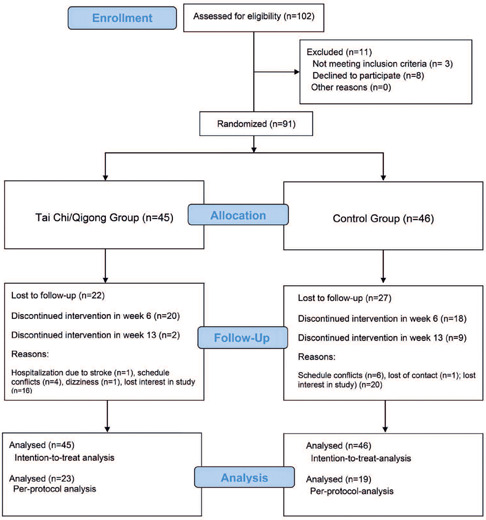
Study flow diagram

All candidates (n=102) were screened, and 91 participants were enrolled in the trial (Figure 1); ultimately, 42 completed the study (23 in the TCQ Group and 19 in the Control Group). Specifically, 25 and 28 participants in the TCQ and Control Groups, respectively, completed at least 50% of the measurements. The attrition rate was high, but did not differ between groups (overall, 52.75% [n=49]; at 6 weeks, intervention, n=20; control, n=18; at 13 weeks, intervention, n=2; control, n=9; χ^2^ = 4.092; df = 1; p=0.083).

The mean frequency of TCQ class attendance was 20.16 (SD = 4.98, n=25), corresponding to a mean adherence rate of 77.5%. Moreover, 35% (n=16) of the participants received at least 75% of the TCQ sessions (19.5 h).

The baseline parameters of each group are shown in table 1. The mean age of the participants was 68.3 years (range, 50-91 years). After randomization, the mean age of the TCQ Group was 66.5 years (SD= 7.6), while that of the Control Group was 70.0 years (SD= 10.1). Most participants were married (n=52, 57.14%), non-workers (n=72, 79.12%), and female (total n=73, 80.22%; intervention n=39, 86.67%; control n=34, 73.91%). Most participants had HTN (52.75%, n=48) or DM and HTN (34.07%, n=31), were overweight (BMI = 28.71 kg/m^2^), and reported no anxiety or depression (n=59, 64.84%). None of the participants in either group reported having practiced TCQ or similar exercises prior to this study. Overall, no statistically significant differences were observed between the groups at baseline.

**Table 1:** Demographic and clinical parameters of the study participants

Variables	Total (n=91)	Groups		p value
		Tai chi/Qigong (n = 45)	Control (n=46)	
Demographic variables				
Sex,n(%)				
Female	73 (80.22)	39 (86.67)	34 (73.91)	0.188
Male	18 (19.78)	6 (13.33)	12 (26.09)	
Age, Years (Mean, SD)	68.26 (9.1)	66.5 (7.6)	70 (10.1)	0.620
Ethnicity, n (%)				
Brown, black, or yellow	47 (51.65)	20 (44.44)	27 (58.70)	0.211
White	44 (48.35)	25 (55.56)	19 (41.30)	
Occupation, n (%)				
Non-worker	72 (79.12)	38 (84.44)	34 (73.91)	0.303
Worker	19 (20.88)	7 (15.56)	12 (26.09)	
Education, n (%)				
Illiterate	4 (4.4)	2 (4.44)	2 (4.35)	0.050
Elementary School	50 (54.95)	19 (42.22)	31 (67.39)	
High School	30 (32.97)	18 (40.00)	12 (26.09)	
Higher Education	7 (7.69)	6 (13.33)	1 (2.17)	
Income (Mean, SD)	1,208.92 (976.03)	1,520 (1430.8)	1,432.8 (466)	0.076
Marital status, n (%)				
Non-married	39 (42.86)	19 (42.22)	20 (43.48)	1.000
Married	52 (57.14)	26 (57.78)	26 (56.52)	
Residence, n (%)				
Lives alone	18 (19.78)	12 (26.67)	6 (13.04)	0.121
Lives with others	73 (80.22)	33 (73.33)	40 (86.96)	
Religion, n (%)				
Yes	85 (93.41)	42 (93.33)	43 (93.48)	1.000
No	6 (6.59)	3 (6.67)	3 (6.52)	
Religion (type), n(%)				
Catholic	45 (49.45)	24 (53.33)	21 (45.65)	0.844
Non-catholic	40 (43.96)	18 (40.00)	22 (47.83)	
Without religion	6 (6.59)	3 (6.67)	3 (6.52)	
Health status and habits				
Physical activity habit, n (%)				
Yes	49 (53.85)	28 (62.22)	21 (45.65)	0.142
No	42 (46.15)	17 (37.78)	25 (54.35)	
Physical activity frequency, n (%)				
<3 times per week	18 (19.78)	11 (24.44)	7 (15.22)	0.192
≥3 times per week)	30 (32.97)	17 (37.78)	13 (28.26)	
Physical activity duration, n (%)				
<1 hour	14 (15.38)	6 (13.30)	8 (17.40)	0.128
≥1 hour	35 (38.46)	22 (48.90)	13 (28.30)	
Diagnosed with anxiety and/or depression?, n (%)				
Yes	31 (34.07)	16 (35.56)	15 (32.61)	0.827
No	59 (64.84)	28 (62.22)	31 (67.39)	
Intake of anxiolytic/anti-depressive medication, n (%)				
Yes	24 (26.37)	11 (24.44)	13 (28.26)	0.813
No	67 (73.63)	34 (75.56)	33 (71.74)	
Diagnosis of DM/HTN, n(%)				
DM	12 (13.19)	8 (17.78)	4 (8.70)	0.238
HTN	48 (52.75)	25 (55.56)	23 (50.00)	
DM and HTN	31 (34.07)	12 (26.67)	19 (41.30)	
Anti-diabetic medication, n (%)				
Yes	38 (41.76)	17 (37.78)	21 (45.65)	0.526
No	53 (58.24)	28 (62.22)	25 (54.35)	
Anti-hypertensive medication, n (%)				
Yes	72 (79.12)	33 (73.33)	39 (84.78)	0.206
No	19 (20.88)	12 (26.67)	7 (15.22)	
Smoking habit, n(%)				
Yes	10 (10.99)	3 (6.67)	7 (15.22)	0.316
No	81 (89.01)	42 (93.33)	39 (84.78)	
Alcohol drinking behavior, n (%)				
Yes	20 (21.98)	12 (26.67)	8 (17.39)	0.415
No	71 (78.02)	33 (73.33)	38 (82.61)	
Following a diet for DM/HTN control, n (%)				
Yes	29 (31.87)	15 (33.33)	14 (30.43)	0.943
No	62 (68.13)	30 (66.67)	32 (69.57)	
Perceived health, n(%)				
Very bad or bad	3 (3.3)	0 (0.00)	3 (6.52)	0.303
Neither good nor bad	28 (30.77)	15 (33.33)	13 (28.26)	
Good or very good	60 (65.93)	30 (66.67)	30 (65.22)	
Clinical variables				
SBP(Mean, SD)	125.95 (15.55)	127.1 (17.1)	124.8 (13.9)	0.482
DBP (Mean, SD)	77.03 (10.17)	77.6 (8.8)	76.5 (11.4)	0.630
BMI (Mean, SD)	28.71 (5.99)	27.2 (6.6)	29.4 (6.6)	0.105
FBG (mg/dl) (Mean, SD)	124.11 (52.02)	117.2 (47.1)	130.8 (56.2)	0.213
Mental health variables				
STAI Score (Mean, SD)	38.63 (10.02)	38.44 (8.8)	38.8 (11.2)	0.865
PSS14 Score (Mean, SD)	22.78 (9.41)	24.42 (8.8)	21.17 (9.8)	0.100

χ^2^ and t-student i-tests were used for statistical analysis.

BMI: Body Mass Index; DBP: diastolic blood pressure; FBG: fasting blood glucose; SBP: systolic blood pressure; STAI:

Strait-Trait Inventory; PSS-14: Perceived Stress Scale

### Primary outcome - anxiety (STAI)

**Figure 2:**
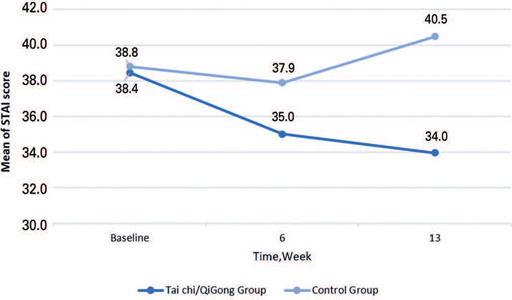
Change in anxiety scores in the Tai Chi/Qigong and Control Groups over 13 weeks

The intention-to-treat analysis (n=40, TCQ Group; n=41, Control Group) showed that after the 13-week intervention, the changes in mean scores on anxiety (STAI scores) were significant for the TCQ Group (6.781 lower -0.360-6.421 = -6.781; Estimate = -6.421; SE = 2.679; 95% confidence interval (95%CI) = [-11.615,-1.224]; p=0.018), which was not observed at week 6 (Table 2). The per-protocol analysis (n=23, TCQ Group; n=19, Control Group) showed a similar result, where after 13 weeks, the changes in mean scores on anxiety (STAI) were significant for the TCQ Group (6.517 lower 0.672-7.189 = -6.517; estimate = -7.189; SE = 2.894; 95%CI= [-12.791,-1.589]; p=0.015) (Table 2). Changes in the STAI scores over time are shown in figure 2.

**Table 2:** Linear mixed-effects model based on the State-Trait Anxiety Inventory score

	Estimate	SE	95%CI	p value
Intent-to-treat Analysis (n=91)				
Intercept	38.804	1.383	36.115-41.494	<0.001
Taichi/Qigong	-0.360	1.966	-4.184-3.464	0.855
Time-Week06	-0.457	1.686	-3.736-2.809	0.787
Time-Week13	2.718	1.959	-1.102-6.513	0.168
Taichi/Qigong*Time-Week06	-2.247	2.445	-6.994-2.491	0.360
Taichi/Qigong*Time-Week13	-6.421	2.679	-11.615-1.224	0.018
Per-Protocol Analysis (n=42)				
Intercept	36.631	2.198	32.376-40.887	<0.001
Taichi/Qigong	0.673	2.970	-5.077-6.423	0.821
Time-Week06	0.737	2.142	-3.407-4.881	0.732
Time-Week13	3.842	2.142	-0.302-7.986	0.076
Taichi/Qigong*Time-Week06	-3.519	2.894	-9.120-2.081	0.227
Taichi/Qigong*Time-Week13	-7.189	2.894	-12.791-1.589	0.015

A linear mixed-effect model was used in the statistical analysis.

95%CI: 95% confidence interval; SE: standard error.

### Secondary outcomes - perceived stress (PSS14)

**Figure 3:**
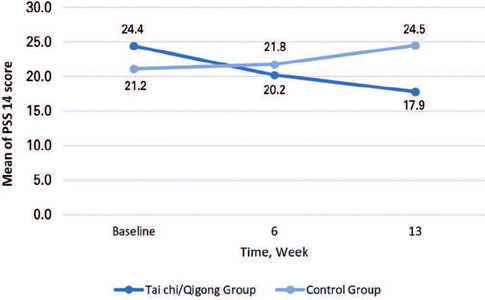
Change in perceived stress scores in the Tai Chi/Qigong and Control Groups over 13 weeks

The intention-to-treat analysis (n=40, TCQ Group; n=41, Control Group) showed lower scores after 13 weeks of TC practice (0.541 lower; 3.238-3.779 = -0.541; estimate = 3.779; SE = 1.653; 95%CI= [0.568,6.981]; p=0.024), which was not observed at week 6. After the 13-week intervention, the changes in the mean scores on stress (PSS14) were significant in the TCQ Group, which were observed from week 6 (1.585 lower; 3.238-4.823 = -1.585; estimate = -4.823; SE = 2.063; 95%CI = [-8.822,-0.824]; p=0.022) and week 13 (6.052 lower; 3.238-9.290 = -6.052; Estimate = -9.290; SE = 2.262; 95%CI= [-13.678,-4.906]; p<0.001) (Table 3). The perprotocol analysis (n=23, TCQ Group; n=19, Control Group) showed that after 13 weeks of intervention, the changes in mean scores on stress (PSS14) were significant for the TCQ Group (-6.657 lower; 1.501-8.158 = -6.657; estimate = -8.158; SE = 2.419; 95%CI = [-12.821,-3.494]; p=0.001) (Table 3). The changes in PSS14 scores over time are depicted in figure 3.

**Table 3:** Linear mixed-effect model based on the PSS14 Score

	Estimate	SE	95% CI	p value
Intent-to-treat Analysis (n=91 )				
Intercept	21.174	1.379	18.486 – 23.861	<0.001
Taichi/Qigong	3.238	1.961	-0.572 – 7.069	0.100
Time-Week06	1.309	1.420	-1.453 – 4.060	0.359
Time-Week13	3.779	1.653	0.568 – 6.981	0.024
Taichi/Qigong*Time-Week06	-4.823	2.063	-8.822 – -0.824	0.022
Taichi/Qigong*Time-Week13	-9.290	2.262	-13.678 – -4.906)	<0.001
Per-Protocol Analysis (n=42)				
Intercept	21.368	2.232	17.033 – 25.703	<0.001
Taichi/Qigong	1.501	3.017	-4.356 – 7.358	0.620
Time-Week06	-0.158	1.784	-3.608 – 3.293	0.929
Time-Week13	3.158	1.784	-0.293 – 6.608	0.080
Taichi/Qigong*Time-Week06	-2.886	2.410	-7.548 – 1.777	0.234
Taichi/Qigong*Time-Week13	-8.158	2.419	-12.821 – -3.494	0.001

A linear mixed-effect model was used in the statistical analysis.

95%CI: 95% confidence interval; SE: standard error.

## DISCUSSION

To the best of our knowledge, this is the first study that investigated the effectiveness of a 13-week TCQ training program (*versus* a waitlist control) in reducing anxiety and perceived stress symptoms in Brazilian middle-aged and older adults with DM and HTN assisted in the primary health care setting. Compared to the Control Group, the TCQ Group showed significantly lower PSS14 scores at 6 and 13 weeks and significantly lower STAI scores after 13 weeks. These results suggest that the implementation of TCQ training in the primary healthcare routine as an adjunct practice to conventional medical treatment of DM and HTN can be an excellent strategy to alleviate symptoms of anxiety and stress and promote the well-being of middle-aged and older adults. In addition, these results are important for individuals with HTN and DM who face difficulties with medication adherence, lifestyle changes (diet and physical exercise), and psychological problems, which negatively impact medical treatment, making control difficult, and accelerating the severity and progression of DM and HTN.^(5,6)^

Although there is a lack of literature regarding the effects of combined TCQ practices on the mental health of diabetic and hypertensive patients in the primary care setting, our findings were consistent with studies developed in other settings (*e.g.,* universities or hospitals) that demonstrated the positive effects of Tai Chi or Qigong practiced independently^(34,35,36,37)^ and combined TCQ^(38)^ on psychological symptoms.

The anxiety reduction observed in this study was also demonstrated in two other studies conducted in individuals with HTN (healthy individuals with stage 1 HTN^(35)^ and older people with essential HTN and mild anxiety^(37)^). These studies used a three-month TC program^(35,37)^ and compared TC training with sedentary life,^(35)^ conventional treatment, and nursing.^(37)^ Tsai et al.^(35)^ reported a reduction in both trait anxiety (-10) and state anxiety (-10.6) in the TC Group. Wang and Ye (2019) showed that the TC Group had a greater reduction in SAS anxiety scores than the Control Group.

Regarding perceived stress reduction, our results agree with those of Chan et al.^(36)^ in a study conducted on hypertensive adults with at least two risk factors for cardiovascular disease. They found a significant reduction in perceived stress in the TC Group when compared with the Control Group (usual care) (-3.22) and brisk walking group (-2.32). James et al.^(38)^ also found similar results in a single-group pilot study demonstrating that eight weeks of TCQ (Tai Chi Easy) significantly reduced perceived stress in middle-aged and older women. However, the authors pointed out the limitations in generalizing the results to other populations, such as the lack of a Control Group and the fact that the study was conducted on a large metropolitan university campus.^(38)^ It is essential to note that not all participants in that study were hypertensive or diabetic, which limits comparisons with our results.

The literature suggests that these improvements in anxiety and stress symptoms can be a result of the link between low-to-moderate physical activity and non-judgment mindfulness characteristics of TCQ training, which influences the reduction of Cortisol and adrenaline, which are hormones related to stress and anxiety^(22,27,51)^ Moreover, the TCQ is usually a group-based exercise that may enhance social support and friendship, thereby impacting the well-being of its practitioners.^(27)^ In our study, the differences in anxiety and stress scores were more significant at 13 weeks, perhaps because the TCQ is a mind-body exercise with a learning curve that requires practice and cultivation to achieve optimal relaxation, calm balance, and wellness.^(35,51)^

### Strengths and limitations

The main strength of this study is its effective approach within the primary care setting and a sample that included a balanced representation of sexes, a broad adult age range (50-91 years old), and diverse socioeconomic backgrounds in Brazil, increasing the external validity of our results for this population. Additionally, it provided primary care providers access to a safe (only one minor adverse event), scalable (virtual), and cost-effective intervention for a holistic/integrative health management plan for patients with DM and HTN. At the end of the study, the participants reported benefits from the program (*e.g.,* improving socialization, mood, physical disposition, body mobility, and reducing body pain), showing that the TCQ is an acceptable and feasible intervention for healthcare services in the community.

This study had some limitations. First, there was a lack of active control for non-specific effects such as social support, attention, treatment contact, and instructor effects.^(52,53)^ Second, the assessors and participants were not blinded to the allocation. Third, the variability in data collection, with in-person interviews for illiterate participants and self-report questionnaires for literate participants, was noted as a limitation because the different methods employed may influence the consistency and reliability of the data, potentially affecting comparability. This approach was chosen to ensure inclusivity, avoiding the exclusion of participants from lower socioeconomic levels. Fourth, the dropout percentage in both groups (52.75%) exceeded the CONSORT recommendations for randomized clinical trials. Although assessments and TCQ classes were conducted within the participants' community to increase accessibility, many participants lost interest and dropped out of the study. Potential explanations for the high attrition rates could be differences in expectations of the program and dissatisfaction with waiting times to start the TCQ classes for the Control Group. Future studies could benefit from brief qualitative interviews at baseline and post-intervention to gain insights from participant feedback, which could inform customized designs for our population. Finally, the lack of long-term follow-up constrains the opportunity to investigate late-emerging outcomes, which are important for detecting psychoemotional-related changes.

## CONCLUSION

In conclusion, the 13-week Tai Chi/Qigong intervention employed in this study effectively reduced anxiety and perceived stress in middle-aged and older Brazilian patients with *diabetes mellitus* and hypertension. These results suggest that Tai Chi/Qigong can be used in primary care centers as an adjunctive therapy to enhance the health of patients with *diabetes mellitus* and hypertension. Future studies should compare Tai Chi/Qigong training with active Control Groups to avoid non-specific effects. Studies should also investigate the feasibility, acceptability, and economic impact of a Tai Chi/Qigong virtual delivery program for patients with *diabetes mellitus* and hypertension in the primary healthcare context.

## Data Availability

Data supporting the findings of this study are available from the corresponding author upon request.
